# Cranial window for longitudinal and multimodal imaging of the whole mouse cortex

**DOI:** 10.1117/1.NPh.9.3.031921

**Published:** 2022-09-23

**Authors:** Marine Tournissac, Davide Boido, Manon Omnès, Yannick Goulam Houssen, Luisa Ciobanu, Serge Charpak

**Affiliations:** a Sorbonne Université, Inserm, CNRS, Institut de la Vision, Paris, France; b Université de Paris, Inserm U1266, Institute of Psychiatry and Neuroscience of Paris, Paris, France; c Université Paris-Saclay, NeuroSpin CEA Saclay, CNRS, Gif-sur-Yvette, France

**Keywords:** chronic cranial window, two-photon imaging, functional ultrasound imaging, BOLD functional magnetic resonance imaging.

## Abstract

**Significance:**

All functional brain imaging methods have technical drawbacks and specific spatial and temporal resolution limitations. Unraveling brain function requires bridging the data acquired with cellular and mesoscopic functional imaging. This imposes the access to animal preparations, allowing longitudinal and multiscale investigations of brain function in anesthetized and awake animals. Such preparations are optimal to study normal and pathological brain functions while reducing the number of animals used.

**Aim:**

To fulfill these needs, we developed a chronic and stable preparation for a broad set of imaging modalities and experimental design.

**Approach:**

We describe the detailed protocol for a chronic cranial window, transparent to light and ultrasound, devoid of BOLD functional magnetic resonance imaging (fMRI) artifact and allowing stable and longitudinal multimodal imaging of the entire mouse cortex.

**Results:**

The inexpensive, transparent, and curved polymethylpentene cranial window preparation gives access to the entire mouse cortex. It is compatible with standard microscopic and mesoscopic neuroimaging methods. We present examples of data on the neurovascular unit and its activation using two-photon, functional ultrasound imaging, and BOLD fMRI.

**Conclusion:**

This preparation is ideal for multimodal imaging in the same animal.

## 1 Introduction

The prominence of blood-oxygen-level dependent functional magnetic resonance imaging (BOLD fMRI) in human imaging results from its noninvasiveness in mapping of brain activation and functional connectivity. However, the extent to which the BOLD fMRI signal actually reports local neuronal activity remains a matter of debate, which has led to the intense development of multiscale and multimodal recording approaches of brain function, in humans and small animals. In rodents, functional ultrasound (fUS) imaging has recently emerged as an efficient alternative to BOLD fMRI for functional brain mapping in anesthetized and awake animals, with millisecond time and mesoscopic spatial resolutions.^1^ fUS detects changes of cerebral blood volume (CBV) of given axial speed with high imaging sensitivity (for reviews, see Refs. 2–4). It can be combined (more easily than BOLD fMRI) with single or multiunit electrodes to correlate the fUS signal and neuronal activity.^5^ However, one drawback of electrodes recording is the impossibility to distinguish the firing of all neuron subtypes. In addition, the chronic placement of metal electrodes interferes with glial physiology and brain elasticity.^6^ This favors the single use of imaging modalities for long-term investigation of brain function, i.e., the combination of fUS or BOLD fMRI with two-photon laser scanning microscopy (TPLSM). TPLSM allows imaging of specific cell types, which can be genetically targeted to express fluorescence reporters of cell activity. As a result, it becomes possible to bridge cellular responses from given neuron subtypes or other cells of the neurovascular unit to mesoscopic signals collected with BOLD fMRI or fUS. This multimodal imaging approach imposes a preparation that complies with all technique limitations: (1) BOLD fMRI acquisition cannot be performed during normal behavioral experiments, but it does not impose any surgery on the mouse head; (2) the fUS signal-to-noise ratio (SNR) is theoretically best without a skull, but is still very good with a thinned-skull or a cranial polymethylpentene (PMP) window^7^ and remains workable through the normal skull and skin;^8^ (3) imaging with TPLSM is best through a cranial glass window but can be achieved through a thinned-skull; (4) both fUS and TPLSM can be performed during behavior in freely moving animals, but glass coverslips prevent fUS and BOLD fMRI. With all these limitations, TPLSM and BOLD fMRI have rarely been combined yet,^9^^,^^10^ the three techniques only once.^11^

**Fig. 1:**
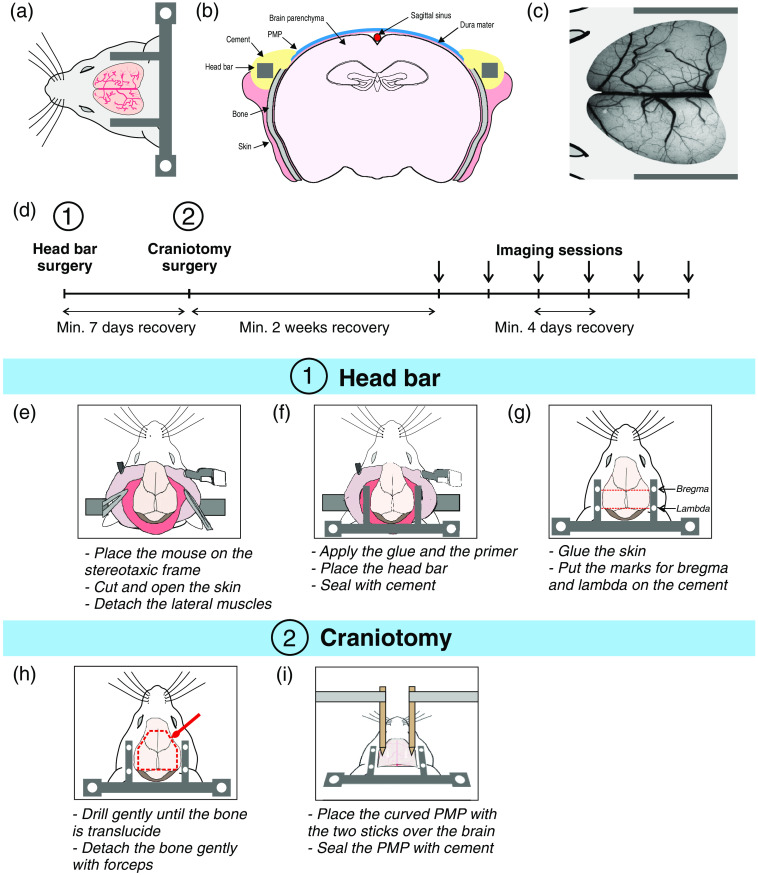
Description of the main steps of the protocol. (a) Schematic of the U-shape head bar placement around the whole cortex craniotomy. (b) The transparent PMP window covers a large part of the neocortex and is sealed with cement to the skull bone. (c) Schematic including a photograph of a whole cortex craniotomy after 40 days. (d) Timeline of the whole procedure. Main steps of the head bar surgery: (e) skull preparation, (f) head bar fixation, (g) stereotaxic landmarks. Main steps of the craniotomy surgery: (h) removal of the skull and (i) placement of the curved PMP.

To fulfill the increasing demand of brain-wide and multimodal imaging, we present here an improvement of the approach we initially developed in the mouse olfactory bulb to bridge cellular activity to fUS and BOLD fMRI signals. We describe the protocol to place a U-shape head bar and a large transparent and curved PMP cranial window that gives access to stable imaging of the entire dorsal neocortex with TPLSM and of the entire brain with fUS and BOLD fMRI [Figs. 1(a)–1(c)]. We provide examples of data acquired with these three imaging modalities, although this preparation is intended to be suitable for a wider range of experimental setups and designs. Indeed, the transparent PMP window should be compatible with most of the common neuroimaging techniques, such as intrinsic optical signal imaging, optical coherence tomography, near-infrared spectroscopy, and confocal microscopy or laser speckle contrast imaging. Finally, our preparation also permits longitudinal imaging to follow the progression of pathological processes.

## 2 Protocol

### 2.1 Implantation of the Whole Cortex Cranial Window

#### 2.1.1 Animals

All animal care and experimentations were performed in accordance with the Institut national de la santé et de la recherche médicale (Inserm) animal care and approved by the ethical committee Charles Darwin (Comité national de réflexion éthique sur l’expérimentation animale – n°5) (protocol number #27135 2020091012114621_v4). Mice were fed *ad libitum* and housed in a 12-h light–dark cycle. Males and females C57/B6 and Thy1-GCaMP6s (GP4.3) mice from 2 to 11 months of age were included. Mice aged more than 2 months were selected because their skull is developed and strong enough to drill easily. We only used mice weighing more than 20 g in our experiments. During imaging sessions, mice were either anesthetized with a mixture of ketamine and medetomidine (100 and 0.5  mg/kg, respectively, intraperitoneal (i.p.)) or sedated with a continuous perfusion of dexmedetomidine (induction with 3% isoflurane and decreased by 0.5% every 5 min down to 0% within 30 min, bolus of dexmedetomidine 0.025  mg/kg subcutaneous (s.c.) at the beginning, and s.c. perfusion of 0.1  mg/kg/h dexmedetomidine during the entire session).

#### 2.1.2 Head bar surgery

Because we noticed that surgical anesthesia limited to 90 min is better tolerated by the rodents, we split the surgery into two different sessions to allocate more time for each of these procedures and nearly abolish mortality due to surgery. We first placed the head bar, waited a minimum of 7 days, and then performed the cranial window surgery [Fig. 1(d)].

A complete list of tools, drugs, and supplies is provided in Table S1 in the Supplemental Material, and a detailed step-by-step protocol is provided in the Supplemental Material. Sterile surgical techniques were done with sterile instruments, surgical supplies, and disinfected stereotaxic frame. The design and dimensions of the U-shape head bar are provided in Fig. S1 in the Supplemental Material and in the following Github repository available at: https://github.com/charpak-lab/U-shape-headbar.

The mouse was injected with dexamethasone (6  mg/kg, s.c.) and buprenorphine (0.3  mg/kg s.c.) 2 h before surgery and then anesthetized with isoflurane (3% induction and 1.5% maintenance). Paw reflexes were carefully verified throughout surgery. Drying of the eyes was prevented via an ocular gel. The animal was placed in a stereotaxic frame with ear bars and an incisor bar to stabilize the head. Body temperature was maintained at 36±0.5°C using a feedback-controlled heating pad with a rectal probe.

Lidocaine was injected under the skin (4  mg/kg s.c.). The hair of the mouse head was removed with commercial depilatory cream that was applied from the neck to the eyes level. The skin was disinfected twice with a betadine solution and cleaned with NaCl buffer. A surgical field was placed over the back of the mouse just below the neck. A midline incision was made in the skin from the neck to the level of the eyes with a scalpel blade, and the skin was maintained on the sides with four clamps. Connective tissue over the skull was removed using a small scalpel blade by gentle scraping from the anterior to the posterior part of the skull. Saline was applied to clean and cool the bone. Then, with small scissors or scalpel, the temporalis muscles were gently detached from the bone all around the skull to laterally leave a 2- to 3-mm clean skull surface to attach the head bar [see Fig. 1(e)]. The area was cleaned with saline and dried with aspiration and air puff. A thin layer of surgical glue was applied to the detached muscles. A layer of primer solution was generously applied to the lateral parts of the skull to enhance adhesion of the photopolymerizable cement to the bone. The U-shape head bar [titanium or plastic (fMRI)] was designed to fit the lateral and posterior part of the skull and our custom head-fixed frame, with holes to fix the head bar to the frame with M3 screws (Fig. S1 in the Supplemental Material). The head bar was first positioned to verify that the detachment of the muscles was deep enough [Fig. 1(f)]. The top of the bar should be aligned to the part where the parietal bone is forming an angle, and the superficial cortical bone should protrude over the top of the bar. After verification of the position of the bar, dental cement was generously applied to the bottom face of the bar. The bar was immediately placed with care to maintain the same horizontal plane as the top of the skull. The cement was hardened with UV light. Then, cement was added to the upper face of the U-shape head bar and in front of the skull to delimitate the external borders of the future craniotomy (Sec. 2.1.3) and, once again, hardened with UV light. Surgical glue was applied all over the exposed skull to protect the bone from deterioration due to contact with air and to suture the skin around the dental cement. Bregma and lambda positions were precisely marked on both sides of the head bar over the dental cement with a bone marker secured to the stereotaxic arm [Fig. 1(g)]. These marks will help to coregister, in a second step, the fUS probe and a camera to record fUS signals from specific coronal slices. The head bar surgery typically lasts less than 45  min. Postoperative care is described in Sec. 2.1.4. Seven days of recovery were respected before the craniotomy.

#### 2.1.3 Cranial window surgery

Because glass is not US-compatible, we replaced the conventional glass coverslip with a transparent PMP (or TPX^®^) sheet (90 to 100  μm thick). This biocompatible, resistant, and flexible material is ideal to preserve the natural curvature of the brain and is easily cut to match the drilled cranial window. Dexamethasone (6  mg/kg, s.c.) was administered 24 h and 1 h before the surgery to prevent brain edema and to reduce inflammation. Buprenorphine (0.3  mg/kg s.c.) was injected as an analgesic 2 h before and 24 h after the surgery. The PMP sheet was shaped a few days before the surgery; it was cut to match the cranial window, cleaned with ethanol 70% and fixed (between two layers of paper with tape) around a plastic tube (22-mm diameter as in Kim et al.^12^) to bend the PMP and mimic the natural curvature of the cortex (see Fig. S2 in the Supplemental Material for detailed information).

The mouse was anesthetized with an i.p. injection of a mixture of ketamine-medetomidine diluted in NaCl (100 and 0.5  mg/kg body mass, respectively) and prepared as described for the head bar surgery. A mixture of 50% air and 50% oxygen was delivered through a nose cone to maintain blood oxygenation during the anesthesia. The skull was disinfected with betadine solution and cleaned with a sterile NaCl buffer. A surgical field was placed over the back of the mouse just below the neck. A motorized drill with a 0.5-mm burr was used to draw a polygon all around the skull [Fig. 1(h)], from lambda to 3-mm rostral to bregma (see Fig. S2 for the size of the cranial window to drill). Drilling of the bone was made carefully and slowly with attention to regularly cool down the bone with fresh artificial cerebrospinal fluid solution (cortex buffer: 125 mM NaCl, 5 mM KCl, 10 mM glucose, 10 mM HEPES, 2 mM ${\mathrm{CaCl}}_{2}$, and 2 mM ${\mathrm{MgSO}}_{4}$ in sterile water, pH 7.4, passed through a sterilization filter, stored at −20°C^13^) and air puff. Drilling was performed slowly until it was possible to see brain vessels through a thin and translucent layer of bone. This part of the surgery usually lasts around 20 min. With small forceps, the softness of the bone was gently checked. When the middle piece of the bone can move, it can be detached. Cortex buffer was generously applied over the bone before removal. It is possible to remove the skull bone in three parts following the skull sutures or in one large piece. Thin and angled forceps were introduced through the thin bone at the bottom right corner of the skull. The bone was slowly pulled up with forceps by doing a lever movement. The homeostatic sponge (gelfoam) wet with cortex buffer was rapidly applied over the dura to prevent bleeding. The upper part of the bone, above bregma, is thicker and has to be removed with more care because the dura seems to be attached stronger to this bone than the rest of the skull. When the bone was removed, attention was paid to remove any dust from the bone on the dura and the remaining part all around the craniotomy because it favors bone regrowth. The piece of PMP was tested and cut if needed to match the shape of the craniotomy and disinfected again with 70% ethanol. A thin layer of cement was applied to the sides of the PMP sheet and polymerized with blue light. The idea is to link this layer of cement to the skull bone with another layer of cement. The PMP was positioned over the brain with cortex buffer between the PMP and the dura. This configuration still allows for small adjustments to correctly fit the craniotomy with the cover. Then, the PMP was mechanically kept in place by two wood or plastic tips secured to the stereotaxic arm [Fig. 1(i)]. The PMP was barely pushed on the brain to keep it close and reduce tissue movement. Then, dental cement was applied all around the drilled bone and the PMP with care to close it. A generous layer of Kwik-Sil was applied over both the PMP window and the cement, and several minutes were respected to let it harden. Kwik-cast (green silicon) could also be used, but the transparency of Kwik-Sil allows to monitor any potential bleeding that can occur in the following days. This step is critical to protect the PMP window and avoid potential damages, such as scratches from other mice or materials present in the cage. The silicon has to be removed before each imaging session by fixing the mouse on the frame, pinching one corner of the silicon with tweezers, and slowly removing it from one side of the cranial window to the other one. The mouse was then injected with atipamezole diluted in NaCl (0.5  mg/kg, s.c.) to antagonize medetomidine-induced muscle relaxation and accelerate recovery of physiological functions. The craniotomy surgery should last less than 90 min. Postoperative care is described in Sec. 2.1.4.

#### 2.1.4 Postoperative care

Additional NaCl (around 200  μL i.p.) was injected if bleeding occurred during the surgery. Each mouse was placed in a recovery heated box for an hour (or until complete awakening) with gel boost. The scoring was performed for 3 consecutive days after the surgery: mice are weighed and behavior, pain signs, and general appearance are evaluated.

### 2.2 Adaptations and Troubleshooting

#### 2.2.1 Adaptation of the environment

Enrichment of the environment is important to maintain proper well-being of experimental animals. We did not need to adapt the environment of the mice after the surgeries because the animals were able to reach the food dispenser and to enter the igloo with the head bar. Plastic wheels were placed in the cage before the head bar surgery to accustom the mice to the support that will be used for awake imaging. It is not necessary (nor recommended by most veterinarians) to isolate the mice after recovery from the surgery, again to preserve their well-being.

#### 2.2.2 Adaptations for magnetic resonance imaging applications

The classical preparation for two-photon and fUS imaging is made with a metal (titanium or stainless steel) head bar for maximum stiffness and photopolymerizable cement for ease of use. Magnetic resonance imaging (MRI) applications require two adaptations. First, the U-shape head bar should be in 3D-printed plastic, and mice should be isolated because the other mice may gnaw the plastic. Second, because the photopolymerizable cement induces artifacts in BOLD imaging, it is substituted with acrylic dental cement.

#### 2.2.3 Surgical considerations and troubleshooting

The chronic preparation described here is complex and requires a minimal surgical formation to avoid a high mortality rate or poor-quality windows. The critical parts of the surgery are the drilling and skull removal. Gentle drilling while regularly cooling the bone reduces reaction of the dura. Because the dura is attached to the bone, delicate skull removal by doing a lever with the tweezers is recommended. It is also possible to remove the bone flap in three different parts following the skull sutures. Rapidly applying wet hemostatic sponge over the dura can help to limit superficial bleedings.

Examples of cranial windows with poor evolution are shown in Fig. S5. Large hemorrhage [see example in Fig. S5(a) in the Supplemental Material] should lead to euthanasia. Reaction from the dura and superficial bleeding usually resorbs within days, but it can accelerate bone regrowth or fibrosis. Decreased imaging quality over time is mainly due to bone regrowth [see Fig. S5(b) in the Supplemental Material]. Note that two-photon imaging can still be done in small spots not covered by the regrowth, while the fUS signal is attenuated by the regrowth [Fig. S5(c) in the Supplemental Material]. Removal and replacement of glass coverslips have been described previously to remove potential bone regrowth arising after months.^14^ However, with a large PMP window, the surgery is very difficult, and it is almost impossible to preserve the dura as it is strongly attached to the regrowth. While sensory-evoked neuronal activity^14^ and arterial dilation^15^ have been shown to be present after dura removal, the impact of dura resection on the glymphatic system has not been considered yet. Overall, practice and care during surgery are keys to improve the long-term quality of the preparation.

Ketamine-medetomidine (or ketamine-xylazine) anesthesia is preferred to isoflurane during craniotomy surgery, because the latter induces larger bleeding due to vasodilation and increased blood flow. Ketamine-medetomidine cocktail implies a supplementation of oxygen (50% oxygen and 50% air), especially during long surgeries, because anesthesia induces hypoxia.

Prevention of infections should be rigorous. Use of antibiotics is not mandatory if sterility is perfectly maintained during the procedure, but it is possible to use a blood-brain-barrier penetrating drug following veterinary recommendations.

Two weeks recovery after craniotomy is recommended before imaging to ensure that superficial damages have been resorbed. Several papers reported increased markers of inflammation following craniotomy using glass windows^13^^,^^14^^,^^16^ lasting at least 2 weeks for glial fibrillary acidic protein immunoreactivity (a marker of astrocytes) and around a week for Iba1 (microglia). In Fig. S4 in the Supplemental Material, we assessed the evolution of brain inflammation below the PMP window (n=2 mice). We implanted a cranial window over a single hemisphere and compared Iba1 staining for microglia in both hemispheres. The craniotomy with PMP-induced microglial activation only in the cortex side covered with the PMP window, an effect that disappeared after 4 weeks, demonstrating that as with glass, brain inflammation fades away with time.

### 2.3 Imaging Methods

#### 2.3.1 Two-photon laser scanning microscopy

TPLSM imaging was performed with a custom microscope using a femtosecond laser with a dispersion compensation module (Mai Tai HP DeepSee; SpectraPhysics, Santa Clara, California) emitting 80-fs pulses at 80 MHz. Laser power was attenuated by an acoustic optical modulator (MT110B50-A1.5-IR-Hk, AA Optoelectronic, Orsay, France). XY scanning was performed with galvanometric scanner (GS) mirrors (8315KM60B; Cambridge Technology, Bedford, Massachusetts). GCaMP6 and Texas Red were excited at 920 nm. The emitted light was collected with a 60X/1.1NA water immersion objective (LUMFLN60XW, working distance 1.5 mm, Olympus, Tokyo, Japan) and was sent to a pair of lenses, coupled into a 2-mm-diameter core polymethyl methacrylate optical ﬁber as previously described.^17^ The collected light was split using a dichroic mirror at 560 nm (FF560-DiO1; Semrock). The green light was ﬁltered with a band-pass filter (FF03-525/50; Semrock). The red light was filtered with a band pass filter (FF01-624/40; Semrock). The signals were each detected with a dedicated gallium arsenide phosphide (GaAsP) photomultiplier tube (H10770PA-40; Hamamatsu Photonics, Japan). In both channels, the laser reflections were blocked with short pass filters (FF01-750/SP; Semrock). Customized Labview (National Instruments, Austin, Texas) software was used to control imaging parameters. The maximum power at the objective was 460 mW. The lateral and the axial point spread functions were 0.35 and 2  μm, respectively. Note that the optical transmittance of the PMP window (80  μm) was >90% from 800 to 1200 nm.

#### 2.3.2 fUS imaging

fUS imaging was performed using an ultrasound scanner (Iconeus One, Iconeus, Paris, France) with a linear ultrasound probe (128 elements, 15 MHz central frequency, Vermon, Tours, France) placed 2 mm above the cranial window. Custom ultrasound sequences were written in Matlab (Mathworks). Ultrasound images of the brain were produced after the backscattered echoes of US plane waves were collected and beamformed. To increase the contrast, the US images were compounded by transmitting several tilted plane waves and adding their backscattered echoes. The compounded sequence resulted in enhanced US images, thereby increasing the sensitivity of the Doppler measurement without aliasing in the mouse brain. In this study, the ultrasound sequence consisted of transmitting 11 different tilted plane waves (from −10  deg to 10 deg in 2 deg increments) with a 5500-Hz pulse repetition frequency (500 Hz frame rate of reconstructed images). Tissue signal was removed from the data using singular value decomposition (SVD) and eliminating the 40 first SVD. The power Doppler (PD) was further ﬁltered with a Butterworth ﬁlter (fifth order). Each voxel signal was obtained by the incoherent temporal average of the blood signal. The voxel size at the focal plane was: 110×100  μm (x and z directions) and 400  μm (y direction, i.e., slice thickness). Data are shown as PD or delta power Doppler (ΔPD) over PD responses (ΔPD/PD), with a baseline of 8 s (from 2 to 10 s). Filtering of axial velocity was done as previously described.^11^

#### 2.3.3 Magnetic resonance imaging recordings

The MR acquisitions were performed on a horizontal 17.2 T small animal MRI scanner (Biospec, Bruker Biospin, Etlingen, Germany) using a volume coil for mouse brain imaging (Rapid Biomedical, Rimpar, Germany). Good B0 homogeneity was ensured through a MAPSHIM correction in the region of interest. fMRI data were acquired using a 2D GE-EPI sequence with the following acquisition parameters: flip angle=40  deg, field of $\text{view}=1.6\times 1.6\;{\mathrm{cm}}^{2}$, in plane $\text{resolution}=200\times 200\;\mu m^{2}$, number of slices = 3, slice thickness=300  μm, echo time = 10 ms, repetition time = 1000 ms, and acquisition time = 16 min. At the end of the fMRI session, high-resolution anatomical images of the same slices were acquired using a rapid acquisition with relaxation enhancement (RARE) pulse sequence: field of view $=1.8\times 1.8\;{\mathrm{cm}}^{2}$, in $\text{plane resolution}=90\times 90\;\mu m^{2}$, echo time = 6 ms, repetition time = 2500 ms, RARE acceleration factor = 6, number of averages = 4, and acquisition time = 5 min 30 s. A thin layer of Kwik-cast (WPI, Sarasota, Florida) was put over the PMP window to reduce air susceptibility artifacts.

### 2.4 Representative Results

In this section, we present multimodal imaging acquisitions performed either with a small PMP chronic window in the mouse olfactory bulb (i.e., published data^11^^,^^18^^,^^19^) or new acquisitions using the whole cortex PMP window described in Sec. 2.1 and Fig. 1.

#### 2.4.1 Microscopic and mesoscopic imaging through a small PMP window in the olfactory bulb

**Fig. 2:**
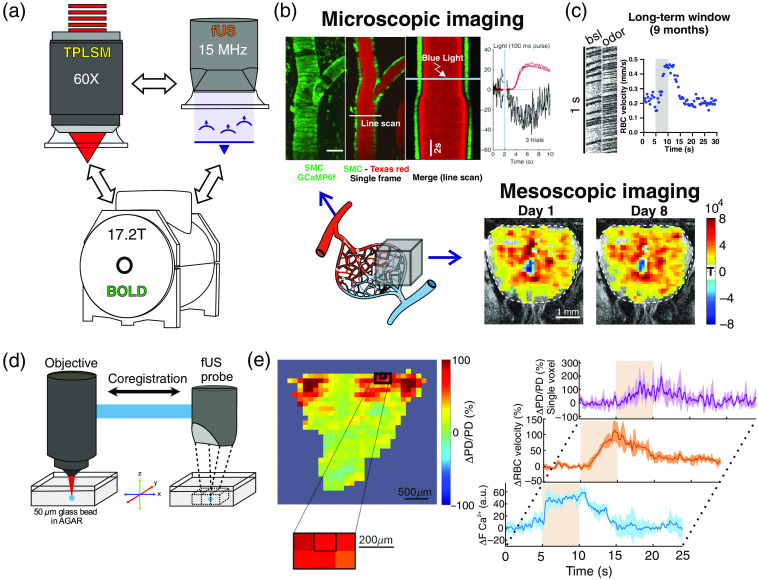
Previous use of PMP for small cranial windows in the olfactory bulb. (a) The same animal can be imaged with two-photon imaging, fUS and BOLD fMRI. (b) Two-photon (microscopic) and fUS (mesoscopic) imaging. Bottom left: schematic of the vascular network, which can be imaged at the microscopic (TPLSM, top) or mesoscopic level (fUS, right). Top: light per se dilates a pial artery labeled with Texas Red. Dilation follows calcium decrease in smooth muscle cells expressing GCaMP6f (scale bar: 30  μm) (modified from Rungta et al.^18^). Bottom: reproducibility of fUS responses (ΔPD) to odor (modified from Boido et al.^11^). (c) Red blood cell velocity response to odor in a capillary imaged (linescan acquisition) 9 months after the implantation of the window (unpublished data). (d) Coregistration of two-photon and fUS imaging systems: a given voxel is centered on a glass bead. (e) A specific voxel, centered on the glomerulus most sensitive to ethyl tiglate (left) shows an fUS response (ΔPD/PD) that mirrors the increase of red blood cell velocity in the glomerulus capillary (modified from Aydin et al.^19^). Average of five responses for fUS and four responses for TPLSM ${\mathrm{Ca}}^{2+}$ imaging and red blood cell velocity.

PMP is an ideal material for multimodal imaging with TPLSM, fUS, and BOLD fMRI in the same animal [Fig. 2(a)]. The use of PMP for fUS imaging was initially reported by Sieu et al.^20^ We then tested the extent to which it was transparent to photons (TPLSM), and whether both techniques could be used in the same mouse with a chronic PMP cranial window. Figure 2(b) shows the use of PMP to investigate a side effect of light in experiments investigating brain function with photoactivation.^18^ In the absence of channelrhodopsin2, light per se (from blue to red light) decreased intracellular calcium in smooth muscle cells, dilating arteriole [Fig. 2(b); top]. The quality of imaging calcium signals or vessel fluorescence through a 250-μm-thick PMP was high. It improved in a following work where we imaged through a 125-μm-thick PMP window and performed sequential imaging with TPLSM, fUS, and BOLD fMRI in the same animal.^11^ Figure 2(b) (bottom right) shows that fUS responses to sensory stimulation (an odor) was highly reproducible through the PMP window, from one week to the other. In fact, the durability of the preparation was such that even with TPLSM, we could image blood flow responses up to 9 months postimplantation [Fig. 2(c)]. The transparency of the PMP window allowed to establish a unique coregistration between fUS and TPLSM with a microscopic precision. By securing the fUS probe to the microscope objective with a custom 3D-printed holder [Fig. 2(d)], we ensured that the center of the fUS voxel was coregistred to the center of the field of view of the microscope imaging plane. We showed that within a given voxel, micro- and mesoscopic vascular responses have similar dynamics [Fig. 2(e)]. In addition, we could compute the first transfer function linking neural calcium and fUS signals.^19^

#### 2.4.2 Multimodal imaging through the optimized whole cortex preparation

**Fig. 3:**
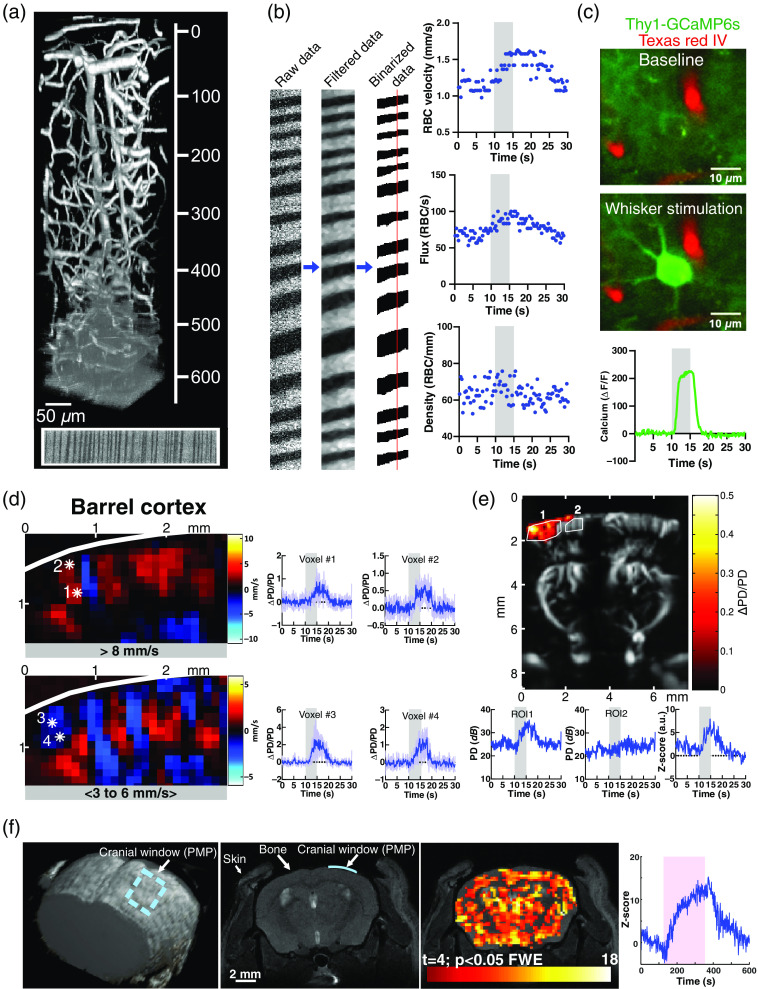
Multimodal imaging through the optimized whole cortex PMP chronic window. (a) 3D reconstruction of the cortical vasculature labeled with Texas Red down to 650  μm and imaged through a 100-μm-thick PMP sheet (ΔZ=1  μm; field of view: 183  μm×169  μm). Bottom: a line scan acquisition at 650  μm. (b) Left: line scan analysis (filtering and binarization) allows to extract red blood cell velocity, flux, and linear density. The vertical red line indicates the site of flow measurement. Right: whisker stimulation (5 s, 5 Hz) increases velocity and flux, not linear density (barrel cortex capillary, 300-μm depth). (c) Calcium response of a pyramidal cell to 5 s whisker stimulation (5 Hz, 400-μm depth) in a Thy1-GCAMP6s mouse. (d) Left: color Doppler map of the barrel cortex in response to a whisker stimulation (5 s, 5 Hz). Colors represent the direction of flow using either a filter for high axial velocities (top image, >8  mm/s) or intermediate axial velocities (bottom image, <3 to 6  mm/s>). Red and blue colors report CBV flowing away or toward the probe, respectively. Right: graphs showing ΔPD/PD responses (mean ± SD of four consecutive whisker responses) of the single voxels indicated by stars (left). Mean contrast-to-noise ratio (CNR) of voxel 1 to 4, respectively; 2.4±0.6, 2.5±0.2, 8.6±4.7, and 4.0±1.4. (e) Top: activation map (ΔPD/PD) superimposed on the Doppler image in response to a single whisker stimulation (5 s, 5 Hz). Bottom: graphs showing the PD response of the activated area (ROI1), the neighboring region (ROI2), and the ratio = (PD ROI1−PD ROI2)/SD ROI1). (f) Left: 3D reconstruction of 2D RARE images. The dotted blue rectangle and the blue line on top of the cortex points to a PMP window (the PMP and the bone are not visible), which small size allowed to reveal the absence of MRI artifact in RARE acquisitions (left and middle images), as well as during a BOLD functional response (Right, T2*-weighted GE EPI acquisition, response to 100% oxygen, 240 s). Right: Z-score of the BOLD signal.

The preparation described in Sec. 2.1 and Fig. 1 presents several improvements adapted for imaging the whole neocortex. The U-shape metal (or plastic for MRI applications) head bar [Figs. 1(a)–1(c)] holds the cranium very tightly while maintaining access to the entire dorsal surface of the cortex, a feature convenient for imaging with both long and short working-distance objectives. In addition to providing long-term solidity (we never experienced any detachment from the skull, even during the first days of training for awake imaging), it offers a good tissue stability for imaging. Figure 3(a) shows that the 100-μm thickness of PMP enables to image the brain vasculature with high quality and to distinguish red blood cells in capillaries down to 650  μm. Filtering and binarization of images acquired with line scan acquisitions allow the extraction of all parameters of capillary blood flow: red blood cells velocity, flux, and linear density at rest and in response to whisker stimulation [Fig. 3(b)]. Although we did not precisely quantify the SNR of calcium signals below a 100-μm-thick PMP window versus a standard glass window, we found that responses to whisker stimulation from pyramids expressing GCaMP6s and located at 400  μm in depth were easily observed [Fig. 3(c)]. Figure S3 in the Supplemental Material shows that the SNR of the vasculature below the PMP window does not decrease with time, at least until 7 weeks postsurgery. The large PMP window offers the possibility to measure fUS responses to neural stimulation in various parts of the neocortex and deeper structures. Figure 3(d) shows an increase in PD (ΔPD/PD) to a 5-s whisker stimulation (5 Hz) in the contralateral barrel cortex. Color Doppler maps for given axial velocities^11^ show CBV flowing above 8  mm/s in vessels, where blood flows away from the fUS probe (red voxels), i.e., in arteries feeding the brain parenchyma. Selecting blood axial velocity between 3 and 6  mm/s reveals CBV flowing in the opposite direction, i.e., veins are draining blood toward the surface (blue voxels). Note that the response SNR allows the detection of responses from single voxels [Fig. 3(d); right]. Figure 3(e) shows the ΔPD/PD activation map in response to a single whisker stimulation superimposed on the Doppler image. Graphs show the corresponding PD response of activated and nonactivated regions of interest (ROIs).

We previously used a small PMP implant for BOLD fMRI using fast low angle shot pulse sequences in the olfactory bulb.^11^ Figure 3(f) shows that the PMP window, provided that it is completely covered by Kwik-cast and combined with a plastic head bar (see Sec. 2.2.2), does not cause artifacts even with gradient-echo echo-planar imaging (GE EPI) sequences, which are very sensitive to differences in magnetic susceptibility (17.2 T magnet). To demonstrate this point, we placed a small PMP window over the cortex of the left hemisphere and collected anatomical and functional MRI data, looking for artifacts below the window and/or at its borders. RARE anatomical acquisitions highlight that PMP does not alter the shape of the cortex surface [Fig. 3(f); left and middle panels]. Similarly, T2*-weighted EPI acquisitions show that exposure of 100% oxygen generates a BOLD acquisition map that matches the anatomical structure of the coronal slice with the same signal increase below the skull bone or the PMP window [Fig. 3(f); right]. We found similar results in the case of the whole cortex craniotomy (Fig. S6 in the Supplemental Material).

## 3 Discussion

Several chronic cranial windows have been described in the literature.^12^^,^^13^^,^^21^^–^^23^ However, the preparation presented here has the advantage of being transparent to light, acoustic waves and devoid of fMRI artifacts, even at an ultrahigh magnetic field (17.2 T). It is, therefore, truly multimodal and compatible with TPSLM, fUS, and BOLD fMRI. The large aperture of the PMP window, its shape adapted to the natural brain curvature, and the head bar fixation granting both cortex-wide imaging and head stability, make this chronic preparation ideal for repetitive imaging sessions in the same animal. Consequently, it can be used for longitudinal investigations in anesthetized or awake animals and has the advantage of reducing the number of animals needed per project.

The main limitation of our procedure is its invasiveness. Preserving the skin and the skull of the mouse is undoubtedly better to maintain brain physiology and microenvironment. Acute removal of the skin alone lowers brain temperature (unpublished). Removing both the skin and the skull lowers brain temperature, but the system adapts with time.^24^ Nevertheless, imaging with a water immersion objective lowers cortical temperature by several degrees, decreasing blood flow and brain oxygenation.^24^ The use of thinned-skull for TPLSM has been a real improvement to reduce light scattering compared with transcranial imaging and inflammation compared with craniotomy with a glass window,^23^ but it remains susceptible to the temperature side effect. Moreover, imaging over days requires gluing a glass coverslip on the bone, preventing bone regrowth, but also imaging with ultrasounds. fUS imaging has also been performed through a completely preserved skull in mice,^8^^,^^25^ but the bone attenuates and distorts acoustic waves^26^ and worsens the image quality. In addition, the reliability over weeks or months of fUS imaging through the skull in the same animal has not been demonstrated yet. A second limitation of the PMP window, which is common to polymers, is that PMP is permeable to gas, and the extent to which it affects brain oxygenation is unknown.

Multimodal imaging is increasingly used in neuroscience to correlate the signals collected with each individual technique. This combination of imaging modalities is necessary to interpret BOLD fMRI or fUS signals.^5^^,^^27^^–^^29^ Using the PMP preparation, we could combine TPLSM and fUS to establish the transfer function between neural activation and the fUS signal at the level of single voxel.^11^ PMP has now been used in numerous fUS studies, in anesthetized or awake rodents,^20^ to optimize ultrasound imaging,^30^ map brain regions involved in the optokinetic reflex,^31^ investigate the neuronal basis of fUS^5^ or the correlation of blood flow with sleep rhythms.^32^ To conclude, the whole cortex preparation will allow to correlate the contribution of all the cell types of the neurovascular unit (endothelial cells, smooth muscle cells, pericytes, and astrocytes) to mesoscopic signals during neurovascular coupling.^33^^–^^38^

## Code, Data, and Materials Availability

The data used in the study are available upon reasonable request to the corresponding author. The files related to the design of the U-shape head bar are provided in the following GitHub repository: https://github.com/charpak-lab/U-shape-headbar.

## References

[1] Macé E. et al., “Functional ultrasound imaging of the brain,” Nat. Methods 8(8), 662–664 (2011).1548-7091 10.1038/nmeth.1641 21725300

[2] Deffieux T. et al., “Functional ultrasound neuroimaging: a review of the preclinical and clinical state of the art,” Curr. Opin. Neurobiol. 50, 128–135 (2018). COPUEN 0959-4388 10.1016/j.conb.2018.02.001 29477979

[3] Rabut C. et al., “Ultrasound technologies for imaging and modulating neural activity,” Neuron 108(1), 93–110 (2020). NERNET 0896-6273 10.1016/j.neuron.2020.09.003 33058769 PMC7577369

[4] Brunner C. et al., “A platform for brain-wide volumetric functional ultrasound imaging and analysis of circuit dynamics in awake mice,” Neuron 108(5), 861–875.e7 (2020). 10.1016/j.neuron.2020.09.020 33080230

[5] Nunez-Elizalde A. O. et al., “Neural correlates of blood flow measured by ultrasound,” Neuron 110(10), 1631–1640.e4 (2022). 10.1016/j.neuron.2022.02.012 35278361 PMC9235295

[6] Macé E. et al., “*In vivo* mapping of brain elasticity in small animals using shear wave imaging,” IEEE Trans. Med. Imaging 30(3), 550–558 (2011). ITMID4 0278-0062 10.1109/TMI.2010.2079940 20876009

[7] Brunner C. et al., “Whole-brain functional ultrasound imaging in awake head-fixed mice,” Nat. Protoc. 16, 3547–3571 (2021).1754-2189 10.1038/s41596-021-00548-8 34089019

[8] Tiran E. et al., “Transcranial functional ultrasound imaging in freely moving awake mice and anesthetized young rats without contrast agent,” Ultrasound Med. Biol. 43(8), 1679–1689 (2017). USMBA3 0301-5629 10.1016/j.ultrasmedbio.2017.03.011 28476311 PMC5754333

[9] Cui M. et al., “A proof-of-concept study for developing integrated two-photon microscopic and magnetic resonance imaging modality at ultrahigh field of 16.4 tesla,” Sci. Rep. 7, 2733 (2017). 10.1038/s41598-017-02864-0 28578390 PMC5457450

[10] Desjardins M. et al., “Awake mouse imaging: from two-photon microscopy to blood oxygen level-dependent functional magnetic resonance imaging,” Biol. Psychiatry Cognit. Neurosci. Neuroimaging 4(6), 533–542 (2019). 10.1016/j.bpsc.2018.12.002 30691968 PMC6556427

[11] Boido D. et al., “Mesoscopic and microscopic imaging of sensory responses in the same animal,” Nat. Commun. 10, 1110 (2019). NCAOBW 2041-1723 10.1038/s41467-019-09082-4 30846689 PMC6405955

[12] Kim T. H. et al., “Long-term optical access to an estimated one million neurons in the live mouse cortex,” Cell Rep. 17(12), 3385–3394 (2016). 10.1016/j.celrep.2016.12.004 28009304 PMC5459490

[13] Holtmaat A. et al., “Long-term, high-resolution imaging in the mouse neocortex through a chronic cranial window,” Nat. Protoc. 4(8), 1128–1144 (2009).1754-2189 10.1038/nprot.2009.89 19617885 PMC3072839

[14] Goldey G. J. et al., “Removable cranial windows for long-term imaging in awake mice,” Nat. Protoc. 9(11), 2515–2538 (2014).1754-2189 10.1038/nprot.2014.165 25275789 PMC4442707

[15] Institoris A. et al., “Astrocytes amplify cerebral blood flow elevation to sustained cortical activation in the awake mouse,” Biorxiv, 2020.12.16.422785 (2020).

[16] Koletar M. M. et al., “Refinement of a chronic cranial window implant in the rat for longitudinal *in vivo* two-photon fluorescence microscopy of neurovascular function,” Sci. Rep. 9, 5499 (2019). 10.1038/s41598-019-41966-9 30940849 PMC6445076

[17] Ducros M. et al., “Efficient large core fiber-based detection for multi-channel two-photon fluorescence microscopy and spectral unmixing,” J. Neurosci. Methods 198(2), 172–180 (2011). JNMEDT 0165-0270 10.1016/j.jneumeth.2011.03.015 21458489

[18] Rungta R. L. et al., “Light controls cerebral blood flow in naive animals,” Nat. Commun. 8(1), 14191 (2017). NCAOBW 2041-1723 10.1038/ncomms14191 28139643 PMC5290324

[19] Aydin A.-K. et al., “Transfer functions linking neural calcium to single voxel functional ultrasound signal,” Nat. Commun. 11, 2954 (2020). NCAOBW 2041-1723 10.1038/s41467-020-16774-9 32528069 PMC7290037

[20] Sieu L.-A. et al., “EEG and functional ultrasound imaging in mobile rats,” Nat. Methods 12(9), 831–834 (2015).1548-7091 10.1038/nmeth.3506 26237228 PMC4671306

[21] Heo C. et al., “A soft, transparent, freely accessible cranial window for chronic imaging and electrophysiology,” Sci. Rep. 6, 27818 (2016). 10.1038/srep27818 27283875 PMC4901295

[r22] Wekselblatt J. B. et al., “Large-scale imaging of cortical dynamics during sensory perception and behavior,” J. Neurophysiol. 115(6), 2852–2866 (2016). JONEA4 0022-3077 10.1152/jn.01056.2015 26912600 PMC4922607

[23] Drew P. J. et al., “Chronic optical access through a polished and reinforced thinned skull,” Nat. Methods 7(12), 981–984 (2010).1548-7091 10.1038/nmeth.1530 20966916 PMC3204312

[24] Roche M. et al., “*In vivo* imaging with a water immersion objective affects brain temperature, blood flow and oxygenation,” Elife 8, e47324 (2019). 10.7554/eLife.47324 31397668 PMC6707784

[25] Bertolo A. et al., “Whole-brain 3D activation and functional connectivity mapping in mice using transcranial functional ultrasound imaging,” J. Vis. Exp. 168, e62267 (2021). 10.3791/62267 33720137

[26] Pinton G. et al., “Attenuation, scattering, and absorption of ultrasound in the skull bone,” Med. Phys. 39(1), 299–307 (2012). MPHYA6 0094-2405 10.1118/1.3668316 22225300

[27] Schulz K. et al., “Simultaneous BOLD fMRI and fiber-optic calcium recording in rat neocortex,” Nat. Methods 9(6), 597–602 (2012).1548-7091 10.1038/nmeth.2013 22561989

[r28] He Y. et al., “Ultra-slow single-vessel BOLD and CBV-based fMRI spatiotemporal dynamics and their correlation with neuronal intracellular calcium signals,” Neuron 97(4), 925–939.e5 (2018). NERNET 0896-6273 10.1016/j.neuron.2018.01.025 29398359 PMC5845844

[29] Winder A. T. et al., “Weak correlations between hemodynamic signals and ongoing neural activity during the resting state,” Nat. Neurosci. 20(12), 1761–1769 (2017). NANEFN 1097-6256 10.1038/s41593-017-0007-y 29184204 PMC5816345

[30] Tang J. et al., “Functional ultrasound speckle decorrelation-based velocimetry of the brain,” Adv. Sci. 7(18), 2001044 (2020). 10.1002/advs.202001044 PMC7509671 32999839

[31] Macé É. et al., “Whole-brain functional ultrasound imaging reveals brain modules for visuomotor integration,” Neuron 100(5), 1241–1251.e7 (2018). NERNET 0896-6273 10.1016/j.neuron.2018.11.031 30521779 PMC6292977

[32] Bergel A. et al., “Local hippocampal fast gamma rhythms precede brain-wide hyperemic patterns during spontaneous rodent REM sleep,” Nat. Commun. 9, 5364 (2018). NCAOBW 2041-1723 10.1038/s41467-018-07752-3 30560939 PMC6299136

[33] Bisht K. et al., “Capillary-associated microglia regulate vascular structure and function through PANX1-P2RY12 coupling in mice,” Nat. Commun. 12, 5289 (2021). 10.1038/s41467-021-25590-8 34489419 PMC8421455

[r34] Császár E. et al., “Microglia modulate blood flow, neurovascular coupling, and hypoperfusion via purinergic actions,” J Exp Med 219(3), e20211071 (2022). 10.1084/jem.20211071 35201268 PMC8932534

[r35] Mishra A. et al., “Astrocytes mediate neurovascular signaling to capillary pericytes but not to arterioles,” Nat. Neurosci. 19(12), 1619–1627 (2016). NANEFN 1097-6256 10.1038/nn.4428 27775719 PMC5131849

[r36] Schaeffer S. Iadecola C., “Revisiting the neurovascular unit,” Nat. Neurosci. 24, 1198–1209 (2021). NANEFN 1097-6256 10.1038/s41593-021-00904-7 34354283 PMC9462551

[r37] Rungta R. L. et al., “Vascular compartmentalization of functional hyperemia from the synapse to the pia,” Neuron 99(2), 362–375.e4 (2018). NERNET 0896-6273 10.1016/j.neuron.2018.06.012 29937277 PMC6069674

[38] Longden T. A. et al., “Local IP3 receptor-mediated Ca^2+^ signals compound to direct blood flow in brain capillaries,” Sci. Adv. 7(30), eabh0101 (2021). STAMCV 1468-6996 10.1126/sciadv.abh0101 34290098 PMC8294755

