# Supplementary material for: Cranial window for longitudinal and multimodal imaging of the whole mouse cortex
Source: Neurophotonics. 2022 Sep 23;9(3):031921. doi: 10.1117/1.NPh.9.3.031921 (PMC9500537; doi:10.1117/1.NPh.9.3.031921)
Supplement: Supplementary file 1 [file NPh_009_031921_SD001.pdf]

Supplemental - *Cranial window for longitudinal and multimodal imaging of the whole mouse cortex*

Name	Reference	Source	Usage
		Supplies	
Ocular gel		Lubrithal, Dechra Veterinary Products	Prevent dry eyes
Vaseline	8995727	Cooper, France	To insert the rectal probe
Depilatory cream		Monoprix, France	Remove the fur
Surgical glue	1469SB	Vetbond, 3M Animal Care Product, USA	Close the skin
Primer	25881E	OptiBond FL Prime, Kerr, Italia	Help to seal the cement to the skull
Pipet tips	10660	Sorenson Bioscience, Inc., USA	Glue and primer application
Photopolymerizable cement	595953WW	Tetric Evoflox A1, Ivoclar Vivadent, Liechtenstein	
Burrs for microdrill (0.5 mm)	19007-05	Fine Science Tools, USA	Drill the bone
Air duster	710-893	Bruneau, France	Remove dusts
Polymehtylpentene (TPX) sheet: transparent, 0.125 mm (100 µm thick measured)*	ME311100	Good Fellow, Canada	Coverslip
Hemostatic sponge	GS-310	Gelita-spon standard, Gelita Medical, Germany	Stop and prevent bleadings
Diet Gel recovery	72-06-5022	Clear H2O, USA	Help recovery of the mouse
Kwik-Sil		World Precision Instrument, USA	Protect the window
		General equipment	
Stereotax apparatus	Model 902	Kopf instruments, USA	Hold the mice
Temperature controller	40-90-8D	FHC, USA	Maintain body temperature during the surgery
Stereomicroscope	SZ4060	Olympus, Japan	Visualisation of the sample
Microdrill	K.1070	Foredom, USA	Drill the bone
UV blue light		Bluephase style, Ivoclar Vivadent, Liechtenstein	Polymerize the cement
Heating box	V1200DT	MediHEAT, Peco Services Ltd, UK	Maintain body temperature right after the surgery
Plastic weels and igloo	K3327, K3251	Fast track, Bio-Serv, USA	Enrichment for the mice
Mouse cage bottom and lids	M-BTM-C8, MSX2	Innovive, USA	Housing
Pre-filled water bottle	M-WB-300A	Innovive, USA	Housing
		Drugs	
Betadine	457581	Vetédine, Vetoquinol, France	Disinfect the skin
Ketamine		Ketamine 1000, Virbac, France	Anesthesia
Medetomidine		Domitor, Orion Pharma, France	Anesthesia
Atipamezole		Antidorm, Axience, France	Reversal of medetomidine
Dexamethasone		Dexazone, Virbac, France	Anti-inflammatory
Buprenorphine		Buprecare, Axience, France	Analgesia
Lidocain		Lidor, Axience, France	Local analgesia
Saline		Virbac, France	Dilution of the drugs and hydration

Surgical tools			
Micro retractors	18052-01	Fine Science Tools, USA	Hold skin
#5 Dumont laminectomy forceps	11223-20	Fine Science Tools, USA	Skin and tissue manipulation
Extra-fine scissors	91500-09	Fine Science Tools, USA	Cut skin or muscles
Sichel knife	10073-14	Fine Science Tools, USA	Cut muscles
Fine forceps - Self-closing	11480-11	Fine Science Tools, USA	Hold the PMP sheet
#5 Dumont forceps	11252-20	Fine Science Tools, USA	Fine manipulations
#5-angles Dumont forceps	11253-20	Fine Science Tools, USA	Bone removal
Common supplies			
Cotton swabs	1504	Societé Bailly, France	Apply betadine
Tissues	115-0600	VWR, USA	Clean surgical area
Wood stick		Monoprix, France	Hold the PMP sheet over the brain
Sterile gauze	1022	Laboratoire Sylamed, France	Cover the animal
Insulin syringe, 29G, 0.5mL	324892	BD Medical, USA	Drug injections
5mL eppendorf	30110487	Eppendorf, Germany	Drug dilutions
2.5mL syringe	SS*02SE1	Terumo, Japan	
Ethanol	20820.362	VWR, USA	Clean surgical area

*For more information about the TPX characteristics: https://jp.mitsuichemicals.com/en/special/tpx/properties/

Table S1. List of the tools and supplies.

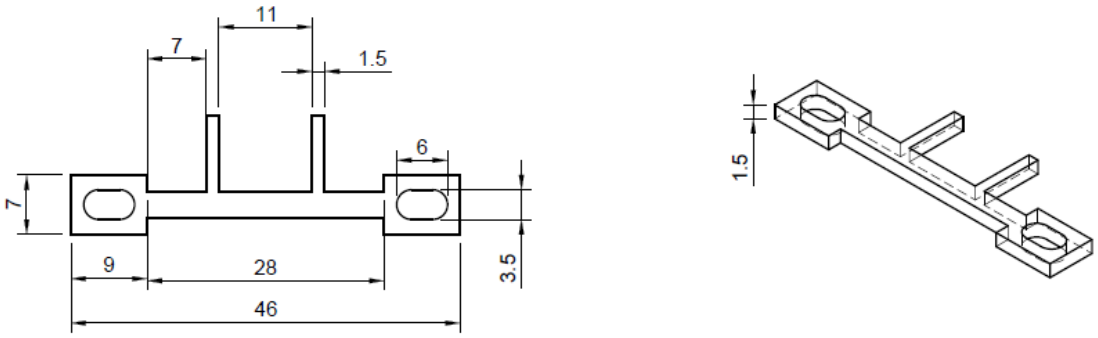

Fig. S1. Design of the U-shape head bar.

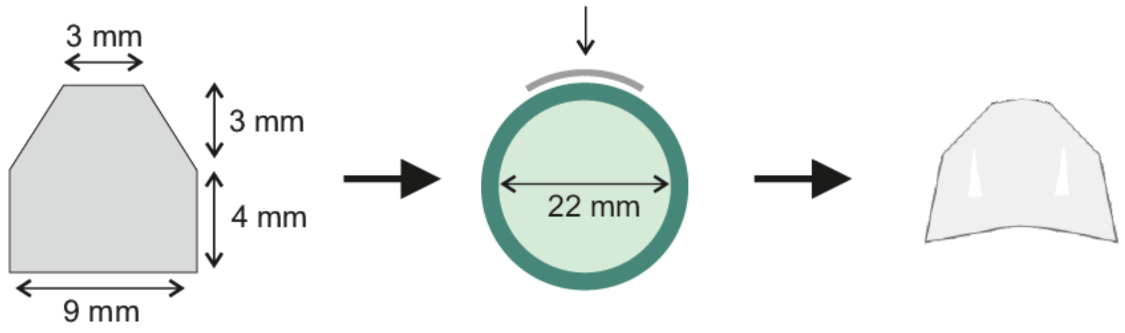

Fig. S2. Template and procedure to curve the PMP sheet.

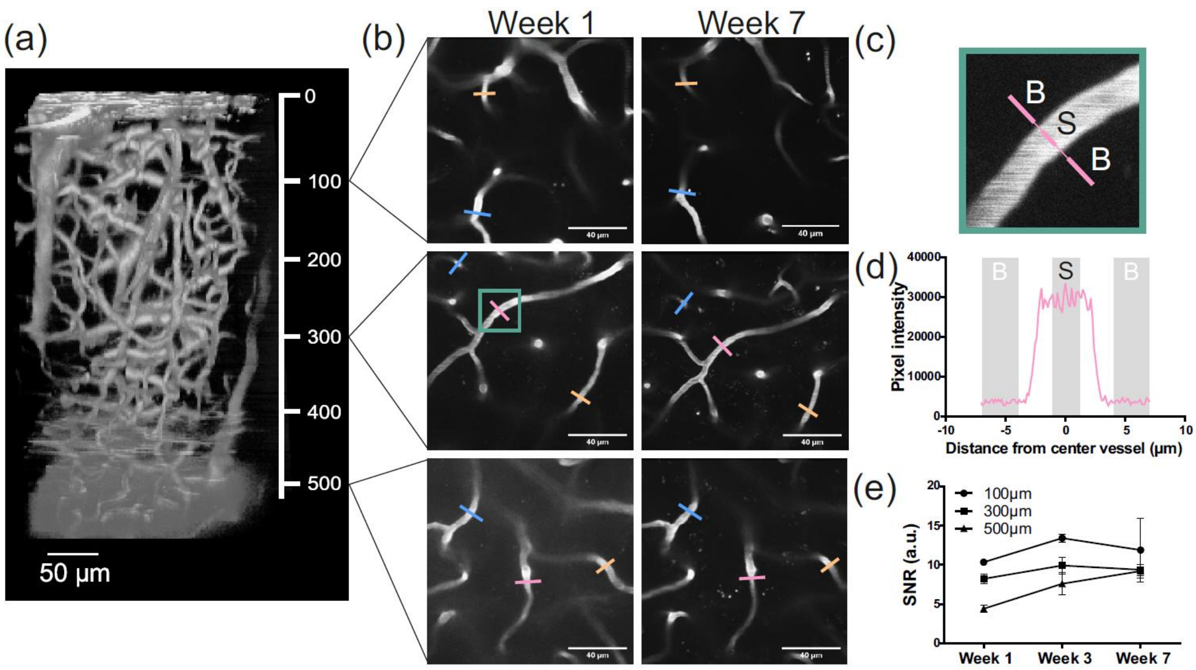

Fig. S3. Longitudinal evaluation of the two-photon imaging SNR.

(a) Z-stack of the vasculature. (b) Three vasculature ROIs were selected at 100, 300 and 500 μm from the brain surface. Fluorescence from 2-3 capillaries per ROIs was monitored over 7 weeks after craniotomy (each image is an average of 8 images, i.e. individual red blood cells are not distinguishable). For all acquisitions, the laser intensity was adapted to maintain an average fluorescence intensity of 30000 a.u within the lumen of vessels. Colored lines show the vessel sites where measurements were done. (c) Representative image of the line portions where the lumen (S) and the background (B) fluorescence was monitored. (d) Pixel intensity plot from (c). (e) Plot showing the SNR evolution over weeks. Note that the SNR at 500 μm depth slightly improved with time.

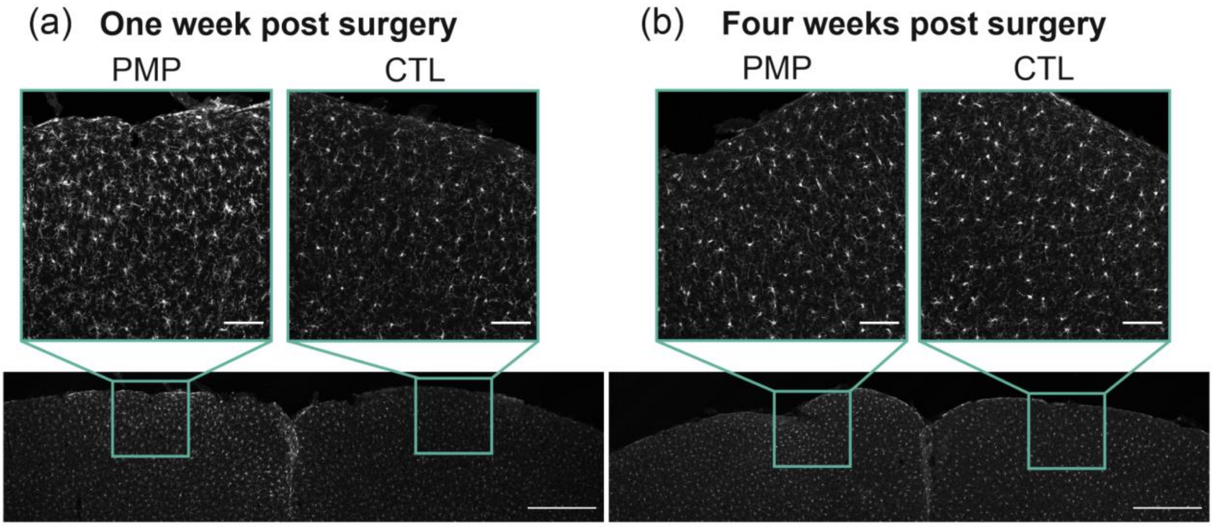

Fig. S4. Iba1 immunoreactivity in the cortex below the PMP window (PMP) and in the contralateral hemisphere (no craniotomy, CTL) after one week (a) and 4 weeks (c) of implantation. Microglia activation is no longer visible 4 weeks after surgery. Scale bar: 100 µm (magnified insets, top) and 500 µm (cortex, bottom).

Immunohistochemistry:

Mice were anesthetized with a mixture of ketamine and medetomidine (100 mg/kg, 0.5 mg/kg respectively, i.p.) and intracardially perfused with 20 mL PBS and then 20 mL PFA 4% in PBS buffer. The brains were post-fixed in PFA 4% overnight at 4°C and then sliced with a frozen microtome in 40 µm-thick sections. Free-floating sections were incubated 1 hour with triton 1% and normal goat serum 4%. Iba1 (1:700, Rabbit, Wako) antibody was incubated overnight at 4°C with agitation in 2% normal goat serum and 0.2% triton. Mounted slices were imaged with a confocal microscope (Zeiss, inverted confocal microscope). The acquisition parameters were the same for the two hemispheres.

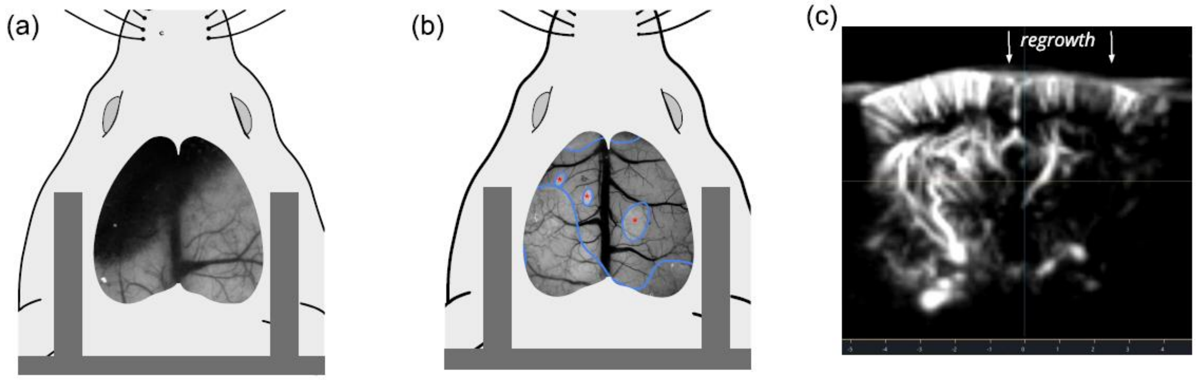

Fig. S5. Examples of cranial windows with poor evolution.

(a) Schematic including a photograph of a hemorrhage in the craniotomy. (b) Schematic including a photograph of a bone regrowth in both hemispheres: it can be sparse (red stars) or “large and cloudy” (outline regions with a blue line). (c) fUS imaging of a mouse brain with bone regrowth over the right hemisphere in which the overall signal is greatly attenuated.

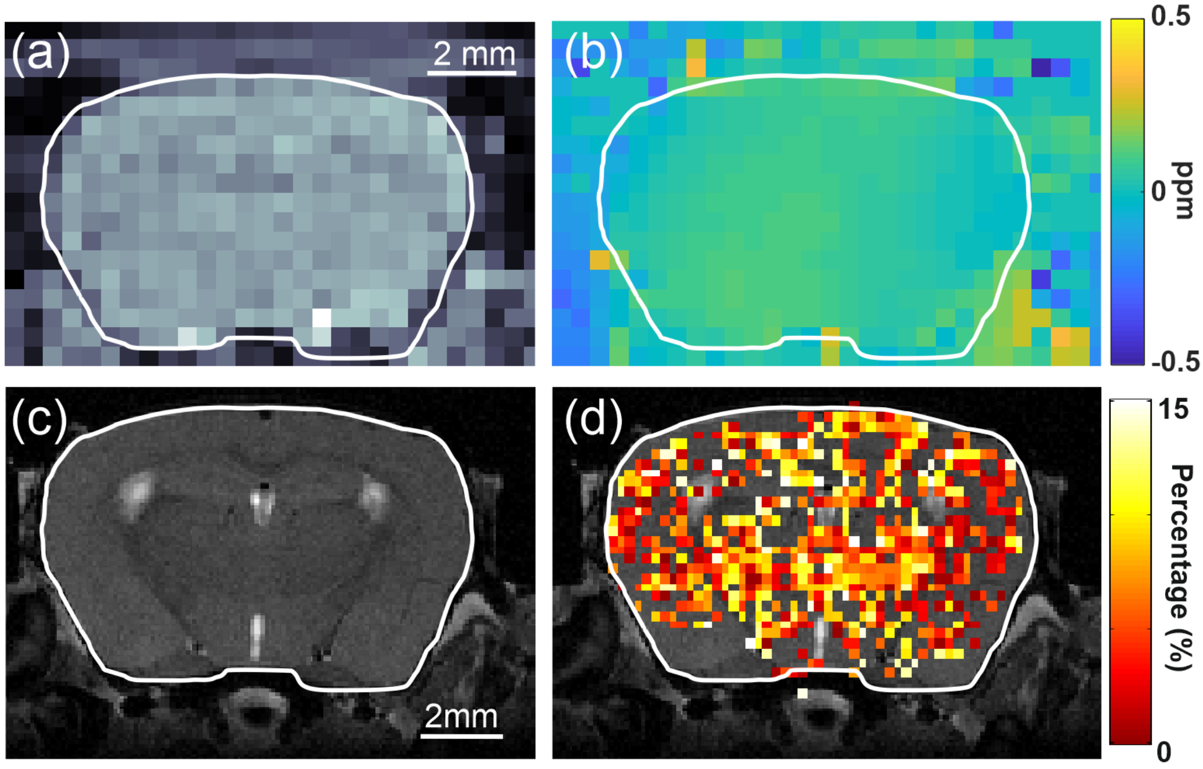

Fig. S6. BOLD fMRI through the whole cortex PMP window.

(a) First echo of B0 acquisition. (b) B0 map. SD = 0.02 ppms. (c) RARE anatomy. (d) BOLD fMRI activation map superimposed over the anatomical image (response to 240 s pure oxygen).

Step-by-step protocol

Head bar surgery. Timing: 45 minutes

1. Inject the mouse with dexamethasone (6 mg/kg, s.c.) and buprenorphine (0.3 mg/kg s.c.) two hours before surgery.
2. Fill the induction chamber with 3% isoflurane and place the animal inside. After two minutes, the breathing rate should be near 1 Hz.
3. Place the animal in the stereotaxic frame with 1.5% isoflurane (maintenance) and place the ear bars and incisor bar to stabilize the head.
4. Maintain body temperature at 36±0.5°C using a feedback-controlled heating pad with a rectal probe and apply ocular gel to prevent eyes drying.
5. Verify lack of paw reflexes by pinching the toes with a tweezer and check carefully throughout surgery.
6. Place a surgical field over the back of the mice just below the neck.
7. Remove the hair of the mouse head by applying commercial depilatory cream from the neck to the eyes level during one minute. Carefully remove the cream and rinse with water.
8. Disinfect the skin twice with a betadine solution and clean with sterile NaCl solution.
9. Inject lidocaine under the skin (4 mg/kg s.c.).
10. Make a midline incision in the skin from the neck to the level of the eyes with a scalpel blade and maintain the skin on the sides with four clamps.
11. Remove the connective tissue over the skull using a small scalpel blade by gentle scraping from the anterior to the posterior part of the skull. Apply saline to clean and cool the bone.
12. Then, with small scissors or scalpel, gently detach the temporalis muscles from the bone all around the skull to laterally leave a 2-3 mm clean skull surface to attach the head bar (see Fig. 1(e)). Clean the area with saline and dry with aspiration.
13. Apply a thin layer of surgical glue to the detached muscles.
14. Generously apply a layer of primer solution to the lateral parts of the skull to enhance adhesion of the photopolymerizable cement to the bone.
15. Position the head bar to verify that the detachment of the muscles is deep enough (Fig. 1(f)). The top of the bar should be aligned to the part where the parietal bone is forming an angle, and the superficial cortical bone should protrude over the top of the bar.
16. Generously apply dental cement to the bottom face of the bar and place it with care to maintain the same horizontal plane as the top of the skull. Harden the cement with UV light.
17. Add cement to the upper face of the U-shape head bar and in front of the skull to delimitate the external borders of the future craniotomy (Section 2.1.3) and, once again, harden with UV light.
18. Apply surgical glue generously over the exposed skull.

    Caution: It is important to protect the skull bone because direct contact with air will lead to deterioration.

19. Suture the skin all around the dental cement.
20. Mark precisely bregma and lambda positions on both sides of the head bar over the dental cement with a bone marker secured to the stereotaxic arm (Fig. 1(g)).

Critical step: These marks will help to record fUS signals from specific coronal slices by coregistering the fUS probe and a camera.

21. Stop isoflurane and place the mouse in a heated box or over a head pad for one hour with gel boost.
22. Inject NaCl (approximately 200 µL i.p.) if bleeding occurred during the surgery.
23. Provide buprenorphine (0.3 mg/kg s.c.) the day after the surgery.
24. Observe and weigh the mouse for three consecutive days after the surgery.

Troubleshooting/Caution: We recommend establishing a scoring grid to evaluate weight, behavior, pain signs and general appearance of the animals and establish a maximal score leading to euthanasia of the animal. In case of dehydration (the skin stays in place when pinching), inject saline solution (200 uL i.p.). In case of weight loss, make sure the diet gel is clean and accessible and add some wet food pellets in the bottom of the cage.

Pause point: Respect at least 7 days of recovery before the craniotomy.

Cranial window surgery. Timing: 90 minutes

25. Inject dexamethasone (6 mg/kg, s.c) 24 hours before the surgery.

Critical step: dexamethasone helps to prevent brain edema and to reduce inflammation.

26. Inject dexamethasone (6 mg/kg, s.c) and buprenorphine (0.3 mg/kg s.c.) two hours before the surgery.
27. Anesthetize the mouse with an i.p. injection of a mixture of ketamine-medetomidine diluted in NaCl (100 mg/kg and 0.5 mg/kg body mass, respectively) and prepare the mouse as described for the head bar surgery.
28. Place the animal in the stereotaxic frame and place the ear bars and incisor bar to stabilize the head.
29. Maintain body temperature at 36±0.5°C using a feedback-controlled heating pad with a rectal probe and apply ocular gel to prevent eyes drying.
30. Verify lack of paw reflexes by pinching the toes with a tweezer and check carefully throughout surgery.
31. Provide a mixture of 50% air and 50% oxygen through a nose cone or a mask to maintain blood oxygenation during the anesthesia.
32. Disinfect the skull with betadine solution and clean with a sterile NaCl buffer.
33. Place a surgical field over the back of the mice just below the neck.
34. Use a motorized drill with a 0.5 mm burr to draw a polygon all around the skull (Fig. 1(h)), from lambda to 3 mm rostral to bregma (see Fig. S2 for the size of the cranial window to drill).

    Critical step : Drill the bone carefully and slowly with attention to regularly cool down the bone with fresh artificial cerebrospinal fluid solution (cortex buffer: 125 mM NaCl, 5 mM KCl, 10mM glucose, 10 mM HEPES, 2 mM CaCl2, 2 mM MgSO4 in sterile water, pH 7.4, passed through a sterilization filter, stored at -20°C[13]) and air puff. Drill slowly until it is possible to see brain vessels through a thin and translucent layer of bone. This part of the surgery usually lasts around 20 minutes.

35. With small forceps, gently check the softness of the bone. When the middle piece of the bone can move, it can be detached. Generously apply cortex buffer over the bone before removal.
36. Introduce thin and angled forceps through the thinned bone at the bottom right corner of the skull. Slowly pull up the bone with forceps by doing a lever movement.

    Critical step: It is possible to remove the skull bone in three parts following the skull sutures or in one large piece. Make sure not to damage the dura during this step. The upper part of the bone, above bregma, is thicker and has to be removed with more care because the dura seems to be attached stronger to this bone than the rest of the skull.

37. Apply the homeostatic sponge (gelfoam) wet with cortex buffer over the dura to prevent bleeding.
38. When the bone is removed, pay attention to remove any dust or remaining part of the bone on the dura and all around the craniotomy.

    Caution: Letting small pieces of bone in the preparation favors bone regrowth.

39. Test the piece of PMP and cut it if needed to match the shape of the craniotomy, and disinfect again with 70% ethanol.
40. Apply a thin layer of cement to the sides of the PMP sheet and make it harden with blue light.

    Critical step: The idea is to link this layer of cement to the skull bone with another layer of cement that will be applied later.

41. Position the PMP over the brain with cortex buffer between the PMP and the dura. This configuration still allows for small adjustments to correctly fit the craniotomy with the cover.
42. Then, mechanically keep the PMP in place with two wood or plastic tips secured to the stereotaxic arm (Fig. 1(i)). Push the PMP slightly on the brain to keep it close and reduce tissue movement.
43. Apply dental cement all around the drilled bone and the PMP with care to close the cranial window.
44. Remove the stereotaxic arms and apply Kwik-Sil (or Kwik-Cast) over the PMP window to protect it and avoid potential damage from other mice in the cage.
45. Inject the mouse with atipamezole diluted in NaCl (0.5 mg/kg, s.c.) to antagonize medetomidine-induced muscle relaxation and accelerate recovery of physiological functions.
46. Inject additional NaCl (around 200 µL i.p.) if bleeding occurred during the surgery.
47. Place the animal in a recovery heated box (or over a heating pad) for an hour or until complete awakening with gel boost.
48. Inject dexamethasone (6 mg/kg, s.c) and buprenorphine (0.3 mg/kg s.c.) the day after the surgery.
49. Perform the scoring for three consecutive days after the surgery the same way as for the head bar surgery.
